# Apoptotic Phosphorylation of Histone H3 on Ser-10 by Protein Kinase Cδ

**DOI:** 10.1371/journal.pone.0044307

**Published:** 2012-09-12

**Authors:** Choon-Ho Park, Kyong-Tai Kim

**Affiliations:** 1 Division of Molecular and Life Science, Department of Life Science, Pohang University of Science and Technology, Pohang, Republic of Korea; 2 Division of Integrative Bioscience and Biotechnology, Pohang University of Science and Technology, Pohang, Republic of Korea; German Cancer Research Center, Germany

## Abstract

Phosphorylation of histone H3 on Ser-10 is regarded as an epigenetic mitotic marker and is tightly correlated with chromosome condensation during both mitosis and meiosis. However, it was also reported that histone H3 Ser-10 phosphorylation occurs when cells are exposed to various death stimuli, suggesting a potential role in the regulation of apoptosis. Here we report that histone H3 Ser-10 phosphorylation is mediated by the pro-apoptotic kinase protein kinase C (PKC) δ during apoptosis. We observed that PKCδ robustly phosphorylates histone H3 on Ser-10 both *in vitro* and *in vivo*. Ectopic expression of catalytically active PKCδ efficiently induces condensed chromatin structure in the nucleus. We also discovered that activation of PKCδ is required for histone H3 Ser-10 phosphorylation after treatment with DNA damaging agents during apoptosis. Collectively, these findings suggest that PKCδ is the kinase responsible for histone H3 Ser-10 phosphoryation during apoptosis and thus contributes to chromatin condensation together with other apoptosis-related histone modifications. As a result, histone H3 Ser-10 phosphorylation can be designated a new ‘apoptotic histone code’ mediated by PKCδ.

## Introduction

Post-translational modifications of histone, including phosphorylation, acetylation, methylation, ubiquitination, sumoylation, and adenosine diphosphate ribosylation, play important roles in the regulation of various cellular processes, such as gene expression, DNA replication and repair, chromatin condensation, chromosome segregation, and apoptotic cell death [1]–[4]. Histone tails are the primary sites of these various types of post-translational modifications and both determine the interaction between histones and other proteins and regulate chromatin structure [5]. The N-terminal tail of histone H3 is able to undergo several different types of epigenetic modification that influence cellular processes [6]. There are several well-characterized residues for phosphorylation within the N-terminal tail of histone H3: Thr-3, Ser-10, Thr-11, and Ser-28. Notably, the phosphorylation of histone H3 on Ser-10 in the N-terminal tail is essential for cell cycle progression and chromosome condensation during mitosis and meiosis [7]. A correlation between histone H3 Ser-10 phosphorylation and chromatin condensation during mitosis has been extensively studied [6], [8]. However, phosphorylation of histone H3 on Ser-10 also occurs when cells are exposed to diverse death stimuli [9], [10]. Although it has been reported that there is a correlation between histone H3 Ser-10 phosphorylation and chromosome condensation during apoptosis, the cellular effects of such phosphorylation remain elusive, and it has not been clearly elucidated whether this phosphorylation contributes to apoptotic cell death [11], [12]. Additionally, there are no reports of specific protein kinases responsible for the phosphorylation of histone H3 on Ser-10 during apoptosis to date.

Protein kinase C (PKC), a family of serine/threonine protein kinases, is activated by various stimuli and involved in cellular processes, such as proliferation, survival, differentiation, and apoptosis [13], [14]. The PKC family is comprised of at least 11 isozymes (α, βI, βII, γ, δ, ε, ζ, η, θ, ι/λ, and μ) categorized according to activators, tissue distribution, and substrates. These PKC isozymes have been classified into three broad groups: i) the classical PKCs (cPKCs: PKCα, PKCβI, PKCβII, and PKCγ), which are calcium-dependent and activated by diacylglycerol (DAG); ii) the novel PKCs (nPKCs: PKCδ, PKCε, PKCη, and PKCθ), which are calcium-independent and activated by DAG; and iii) the atypical PKCs (aPKCs: PKCζ and PKCι), which are calcium-independent but not activated by DAG [15]. PKCδ, a member of the nPKC family, is activated by diverse stimuli, including anti-cancer drugs, ionizing radiation, reactive oxygen species, growth factors, and cytokines [16]. In fact, many apoptotic stimuli can induce PKCδ translocation to mitochondria, resulting in cytochrome C release and caspase-3 activation, as well as the generation of a constitutively active PKCδ catalytic fragment [17]. Upon exposure of cells to DNA-damaging agents, PKCδ undergoes caspase-3-mediated cleavage, resulting in the generation of catalytic fragment of PKCδ. In most cells, catalytic fragment of PKCδ is associated with apoptotic cell death. Ectopic expression of the catalytic fragment of PKCδ is sufficient to induce apoptotic cell death [18], [19]. In addition, PKCδ-deficient cells are resistant to stresses induced by several stimuli, suggesting that PKCδ is crucial for apoptotic cell death [20]. Therefore, PKCδ is generally considered a growth inhibitory or pro-apoptotic kinase in mammalian cells.

Here, we show that phosphorylation of histone H3 on Ser-10 during apoptosis is mediated by the pro-apoptotic kinase PKCδ. It is responsible for the phosphorylation of histone H3 on Ser-10 *in vitro* and *in vivo.* Ectopic expression of catalytically active PKCδ robustly induced phosphorylation of histone H3 on Ser-10 and results in apoptotic cell death. We also demonstrate that activation of PKCδ is required for histone H3 Ser-10 phosphorylation during apoptosis and thus contributes to chromatin condensation.

## Results

### PKCδ Specifically Phosphorylates Histone H3 on Ser-10 *in vitro*

**Figure 1:**
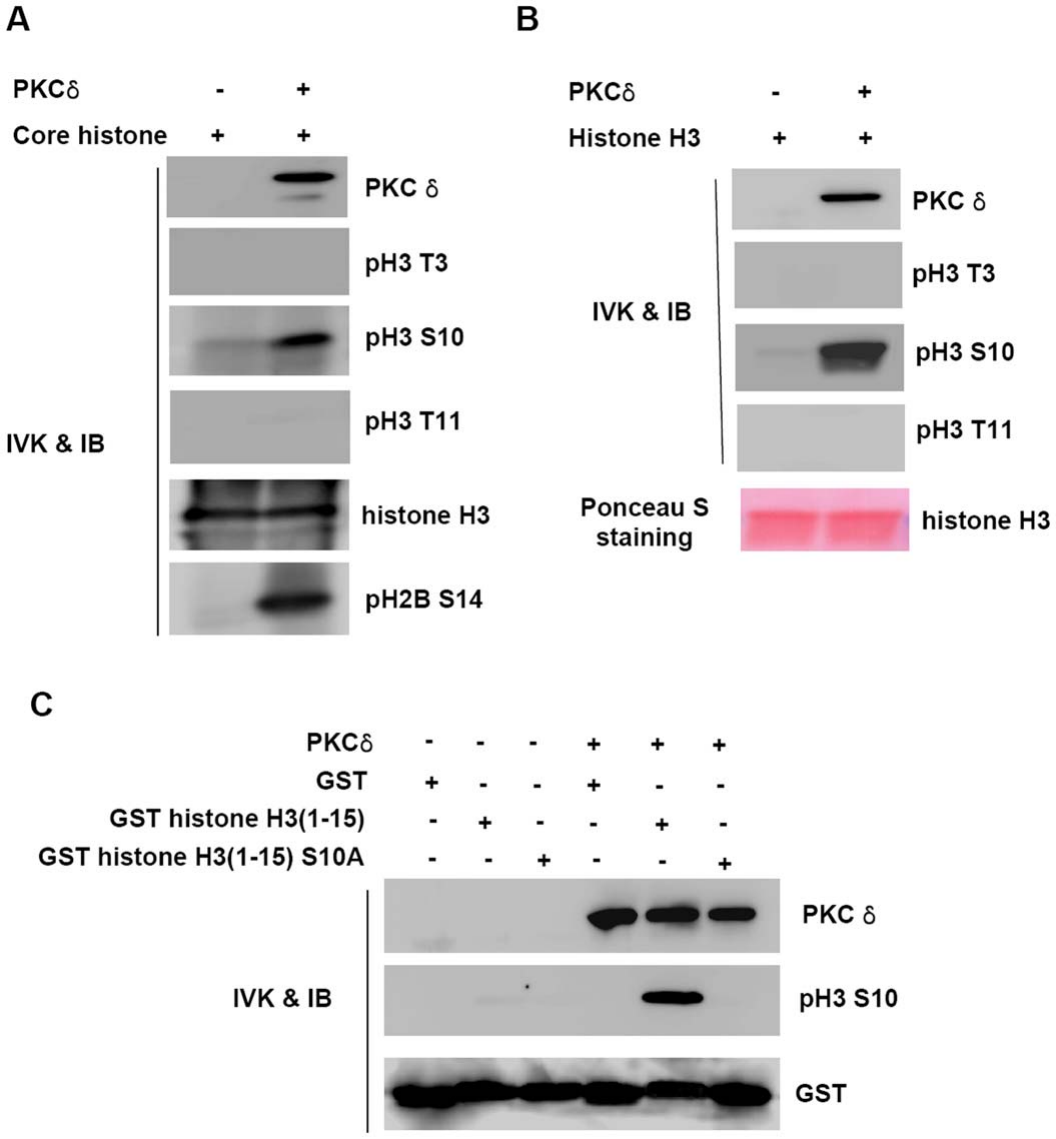
PKC δ phosphorylates Ser-10 of histone H3 *in vitro.* (**A**) **and** (**B**), Recombinant PKCδ was incubated with ATP and core histone (A) or histone H3 (B). After the *in vitro* kinase(IVK) assay, the samples were analyzed by immunoblotting (IB) with anti-PKCδ, anti-phospho histone H3 Thr 3 (pH3 T3), anti-phospho histone H3 Ser 10 (pH3 S10), anti-phospho histone H3 Thr 11 (pH3 T11), anti-phospho histone H2B Ser 14 (pH2B S14) or anti-histone H3 antibody. (**C**) Recombinant GST, GST-histone H3(1–15) or GST-histone H3(1–15, S10A) proteins were incubated with recombinant PKCδ. Immunoblottings were probed with anti-phospho histone H3 Ser 10 (pH3 S10) or anti-GST antibodies.

In our attempt to find a novel histone kinase, we identified that PKCδ robustly phosphorylates histone H3 in a protein kinase assay using radioactive adenosine triphosphate (data not shown). To elucidate whether PKCδ is involved in the modification of the N-terminal tail of histone H3, we performed an *in vitro* kinase assay and analyzed specific phosphorylation by immunoblotting with several anti-phosphohistone H3-specific antibodies. The N-terminal tail of histone H3 is subject to diverse phosphorylation, including Thr-3 by haspin and VRK1, Seri-10 by Aurora B and VRK1, Thr-11 by Dlk/Zip kinase, and Ser-28 by Aurora B [21]–[25]. As shown in Figure 1A, PKCδ specifically phosphorylated free core histones on Ser-10 of histone H3. As expected, the Ser-14 residue of histone H2B was also phosphorylated by PKCδ [26]. Likewise, PKCδ also phosphorylated Ser-10 when purified histone H3 was used as a substrate (Figure 1B). Importantly, the ability of PKCδ to phosphorylate histone H3 on Ser-10 is completely abolished when the residue is mutated to alanine (Figure 1C).

In contrast, other known phosphorylation sites located in the N-terminal tail of histone H3, Thr-3 and Thr-11, were unaffected by PKCδ when either core histone or free histone H3 were used as substrates (Figure 1A and B). We also tested whether Ser-28 of histone H3 is also phosphorylated by PKCδ, because both Ser-28 and Ser-10 are located immediately after the same amino acid motif–ARKS–in the tail of histone H3. As shown in Figure S1, PKCδ phosphorylates Ser-28 of histone H3 when both core histone and free histone H3 were used as substrates. PKCδ phosphorylates histone H3 at Ser-10 and Ser-28 *in vitro* simultaneously as does Aurora B.

### Catalytically Active PKCδ Phosphorylates Histone H3 on Ser-10 in Cells

**Figure 2:**
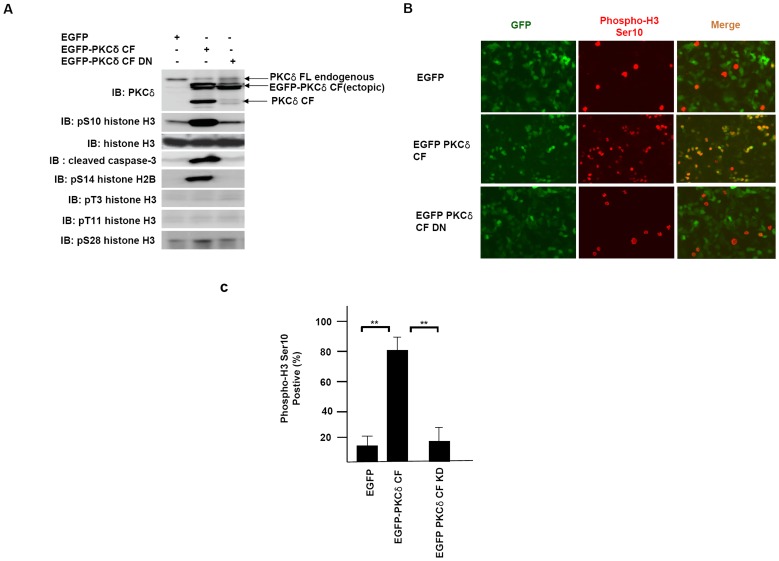
Ectopic expression of PKCδ induces Ser-10 phosphorylation of histone H3. (**A**) HEK 293A cells were transfected with EGFP vector, EGFP-PKCδ catalytic fragment (CF) and EGFP-PKCδ dominant negative form of catalytic fragment (CF DN), respectively.At 24 hours after transfection, cell lysates were subjected to immunoblot analysis with indicated antibodies. (**B**) Immunofluorescence staining of HEK293A cells transfected with PKCδ constructs. HEK293A cells were transfected with EGFP vector, EGFP-PKCδ CF and EGFP-PKCδ CF DN, respectively. Phosphorylation of histone H3 Ser 10 was monitored with specific antibody. (**C**) Kinase activity of PKCδ is required for histone H3 Ser 10 phosphorylation. Kinase-dead PKCδ CF DN was unable to phosphorylate histone H3 on Ser 10. Numbers of positive cells with anti-phospho histone H3 Ser 10 staining were calculated with means±SD value of three independent experiments. **, P<0.01.

To explore the physiological role of PKCδ in mediating histone H3 Ser-10 phosphorylation, we transfected HEK293T cells with plasmids expressing either a constitutively active catalytic fragment (CF) or a kinase-dead dominant-negative (CF-DN) PKCδ mutant (Figure 2A). Ectopic expression of PKCδ CF dramatically increased the level of phosphorylation on Ser-10 of histone H3, whereas there was no substantial change in cells expressing PKCδ CF-DN (Figure 2A). Catalytically active PKCδ significantly enhances the level of phosphorylation on Ser-14 of histone H2B, a typical marker of chromatin condensation during apoptosis. Under the above conditions, apoptotic cell death signaling was evident from the increased amount of cleaved caspase-3 (Figure 2A). In accordance with the *in vitro* kinase assay shown in Figure 1 A and B, there were no remarkable changes in phosphorylation on Thr-3 or Thr-11 of histone H3 when HEK293A cells were transfected with catalytically active PKCδ (Figure 2A). However, it is noteworthy that there were no significant increases in the levels of phosphorylation on Ser-28 of histone H3 in PKCδ-overexpressing cells (Figure 2A). These data indicate the possibility that histone H3 Ser-28 is not the main phosphorylation site by PKCδ under physiological conditions.

To demonstrate whether PKCδ triggers the phosphorylation of histone H3 on Ser-10 during apoptosis, we stained cells with phospho-H3 Ser-10-specific antibody. Cells transfected with PKCδ CF induced remarkable histone H3 Ser-10 phosphorylation but not in PKCδ CF-DN- or mock vector-transfected cells (Figure 2B). Strikingly, catalytically active PKCδ elicited histone H3 Ser-10 phosphorylation in greater than 80% of transfected cells (Figure 2C). Collectively, these observations suggest that activation of PKCδ is sufficient to promote the phosphorylation on Ser-10 of histone H3 during apoptotic cell death.

### Phosphorylation of Histone H3 on Ser-10 by PKCδ during Apoptotic Cell Death

**Figure 3:**
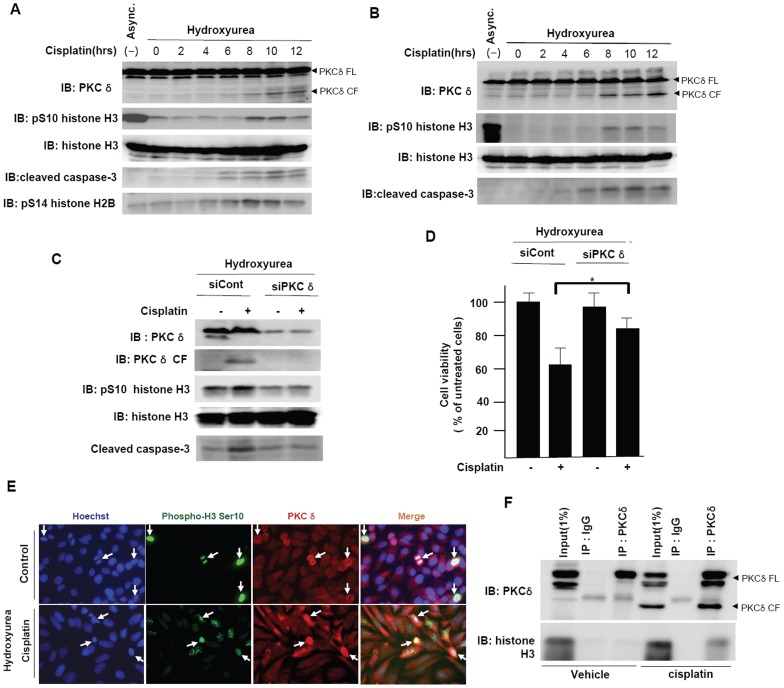
Phosphorylation of histone H3 on Ser10 in apoptotic cell death. (**A**) **and** (**B**) Jurkat (A) or HeLa (B) cells were treated with 1 mM hydroxyurea for 24 hours and then treated with cisplatin (50 µM) for the incubated times. Whole cell lysates were analyzed by immunoblotting with indicated antibodies. (**C**) Jurkat cells transfected with control siRNA or PKCδ siRNA were untreated or treated with cisplatin (50 µM) for 12 hours. The samples were analyzed by immunoblotting with indicated antibodies. (**D**) Jurkat cells were transfected scramble control siRNA or PKCδ siRNA for 24 hours. Then cells were treated with cisplatin (50 µM) for 9 hours. Cell viability was measured by MTT assay. *, P<0.05. (**E**) HeLa cells were treated with 1 mM hydroxyurea for 24 hours and then treated with cisplatin (50 µM) for 12 hours, and anti-PKCδ or phospho-histone H3 Ser 10 staining cells were detected using immunofluorescence microscope. (**F**) Whole cell extracts from Jurkat cells treated with vehicle(DMSO) or cisplatin (50 µM) were immunoprecipitated with anti-PKCδ antibody, and the precipitates were immunoblotted with antibodies to PKCδ or histone H3.

To investigate the physiological relevance between histone H3 Ser-10 phosphorylation and apoptotic cell death, we tested whether the histone modification occurred in cells during DNA damage-induced apoptosis. To examine the role of histone H3 Ser10 phosphorylation in apoptotic cell death, it is necessary to exclude mitotic histone H3 Ser10 phosphorylation. To do this, we synchronized the cells to G1 to exclude the phosphorylation of H3 on Ser10 in mitosis. Jurkat cells (Figure 3A) or HeLa cells (Figure 3B) were pretreated with hydroxyurea for 24 hrs before the addition of cisplatin to elicit DNA damage-induced apoptosis. As expected, cells arrested in the G1 phase after treatment with hydroxyurea showed decreased levels of phosphorylation on Ser-10 of histone H3 (Figure 3A and B). After cell cycle synchronization, the treatment of cells with cisplatin triggered the induction of major apoptotic markers, such as cleaved caspase-3 and PKCδ CF. Additionally, the phosphorylation of histone H3 on Ser-10 was also increased, suggesting that PKCδ is the kinase responsible for the phosphorylation of histone H3 on Ser-10 that occurs during programmed cell death (Figure 3A and B). To confirm whether PKCδ is responsible for histone H3 Ser-10 phosphorylation during DNA damage-induced apoptosis, we knocked down PKCδ by transfection of Jurkat cells with PKCδ siRNAs. Down-regulation of PKCδ abrogated histone H3 Ser-10 phosphorylation induced by cisplatin (Figure 3C). Moreover, the induction of cleaved caspase-3 was diminished (Fig. 3C). On the basis of MTT assay, we also found that depletion of PKCδ resulted in resistance to apoptotic cell death following cisplatin treatment as well as reduction of histone H3 Ser10 phosphorylation (Fig 3D). These data suggest that PKCδ-mediated histone H3 Ser10 phosphorylation is also crucial in apoptotic cell death. Besides, to investigate whether PKCδ specifically phosphorylates histone H3 Ser10, we tested other PKC isoenzymes. When PKCα and PKCε were depleted, the phosphorylation of histone H3 on Ser10 were not affected by cisplatin treatment, suggesting that this phosporylation is solely induced by PKCδ in DNA damage-induced apoptosis (Figure S2).

To demonstrate the involvement of endogenous PKCδ in the phosphorylation of histone H3 at Ser10 during apoptotic cell death, cisplatin-treated HeLa cells were examined by immunofluoresence microscopy with anti-PKCδ and anti-phosphohistone H3 Ser10-specific antibody after cell cycle arrest induced by hydroxyurea. As shown in Figure 3E (upper panel), cytoplasmic localization of PKCδ was prominent in most untreated control cells. In addition, PKCδ antibody did not stain the mitotic cell chromosomes which was positive for the phospho-histone H3 Ser10 (see arrows), confirming that PKCδ is not associated with mitotic chromosomal condensation. In contrast, when HeLa cells were treated with cisplatin after cell cycle synchronization (Figure 3E, lower panel), activated PKCδ translocated to the nucleus and co-localized with the phospho-histone H3 Ser10 positive cells(see arrows). This immunocytochemical analysis suggests that apoptotic phosphorylation of histone H3 on Ser10 is associated with the activation and translocation of PKCδ in DNA damage-induced apoptosis. To investigate the nuclear translocation and colocalization of PKCδ with phospho-histone H3 Ser 10 in DNA damage-induced apoptosis, we also performed co-immunoprecipitation assay before and after exposing the cells to cisplatin. We could detect endogenous interaction between activated PKCδ and histone H3 only under DNA-damaging conditions, suggesting that they could associate with each other in process of apoptotic cell death (Figure 3F).

**Figure 4:**
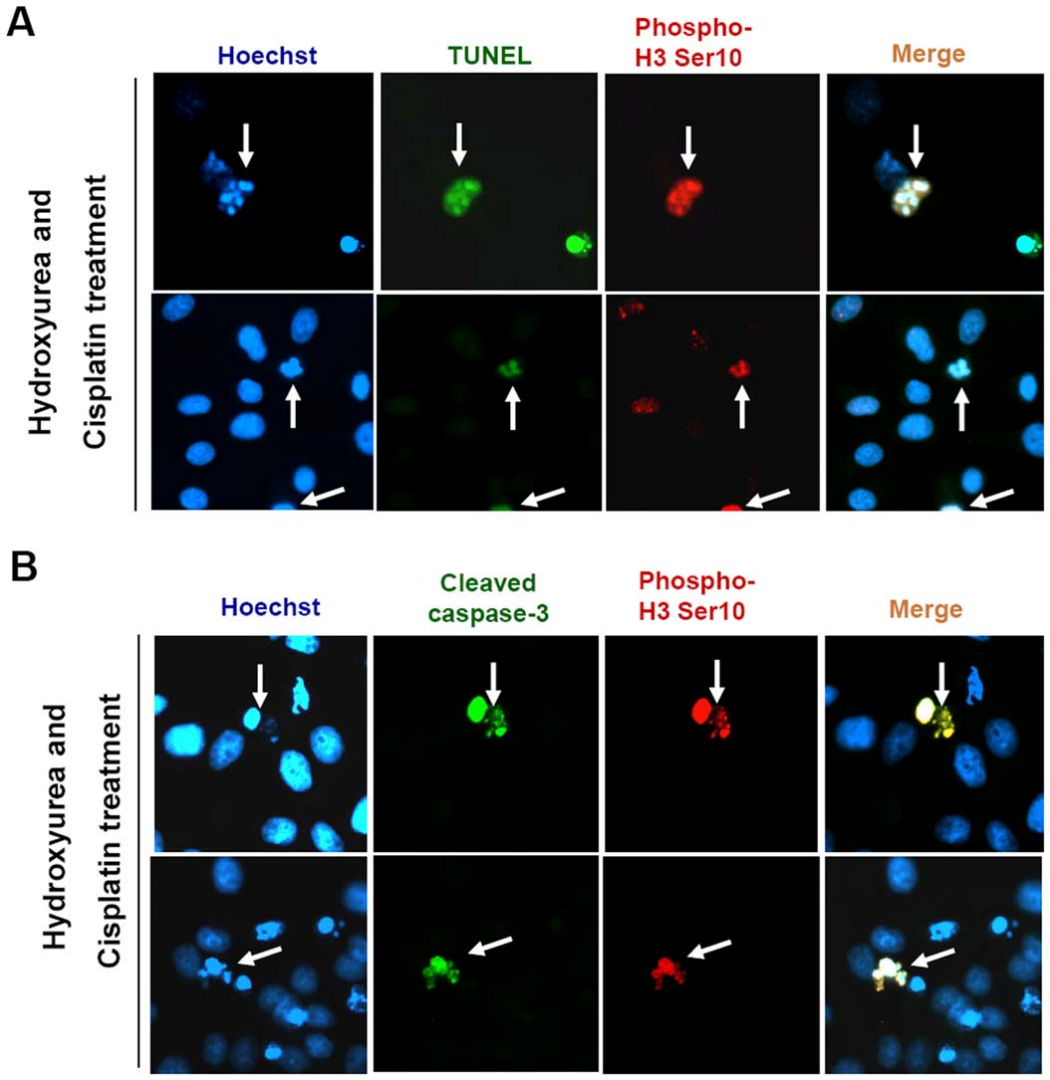
Phosphorylation of histone H3 on Ser-10 in apoptotic cells. (**A**) **and** (**B**) HeLa cells were treated with 1 mM hydroxyurea for 24 hours and then treated with cisplatin (50 µM) for 12 hours, and TUNEL (A), cleaved caspase-3 (B) and phospho-histone H3 Ser 10 staining cells were detected using immunofluorescence microscope. Upper and lower panel images were taken from the same experimental set (A and B).

Furthermore, cisplatin-treated HeLa cells were also analyzed by both terminal deoxynucleotidyl transferase dUTP nick end labeling (TUNEL) assay and immunocytochemical analysis using anti-phosphohistone H3 Ser10-specific antibody. Surprisingly, we found that about 10% of TUNEL-positive cells were also positive for the phosphorylation of histone H3 on Ser10 (Figure 4A and Figure S3). In addition, cleaved caspase 3-positive cells were also labeled with anti-phosphohistone H3 Ser10 antibody (Figure 4B). Hence, these data indicate that PKCδ mediates the phosphorylation of histone H3 on Ser10 during apoptotic cell death.

### PKCδ-mediated Phosphorylation of Histone H3 on Ser-10 during Apoptosis Contributes to Chromatin Condensation

Post-translational modifications of histone proteins have the ability to regulate nucleosome structure. Chromatin condensation and DNA fragmentation are the most prominent nuclear features that occur during apoptotic cell death [27]. According to a previous report, PKCδ promotes histone H2B Ser-14 phosphorylation and subsequent chromatin condensation during apoptosis [26], [28]. Therefore, we hypothesized that PKCδ contributes to chromatin condensation by phosphorylating histone H3 on Ser-10 during programmed cell death.

**Figure 5:**
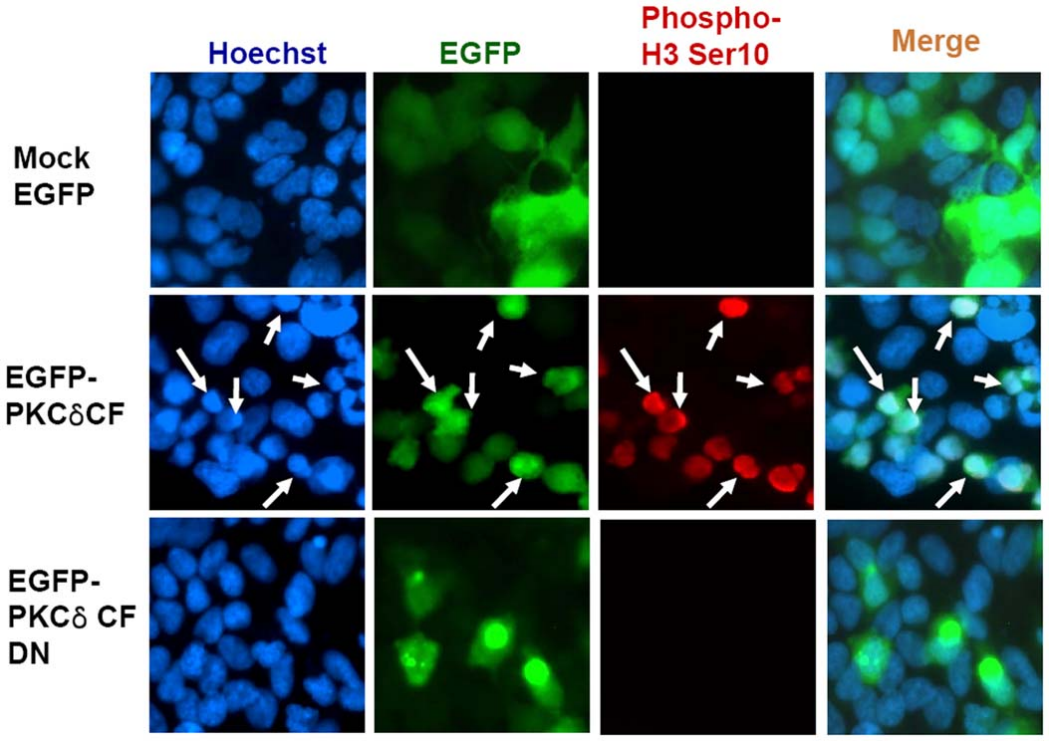
PKCδ-mediated phosphorylation of histone H3 on Ser-10 contributes to apoptotic chromatin condensation. HEK293A cells were transfected with EGFP vector, EGFP-PKCδ CF and EGFP-PKCδ CF DN, respectively. Chromatin condensation was analyzed with fluorescent microscope after Hoechst staining. The condensed nuclei were indicated with white arrows (middle panel).

To test whether activated PKCδ is involved in chromosome condensation during apoptosis, HEK293A cells were transfected with enhanced green fluorescent protein (EGFP), EGFP-PKCδ CF, and EGFP-PKCδ CF-DN plasmids, respectively. The transfected cells were labeled with anti-phosphohistone H3 Ser-10 antibody, and the nuclei were stained with Hoechst 33342. As shown in Figure 2B, cells transfected with PKCδ CF also stain positive for histone H3 Ser-10 phosphorylation. These cells also contain shrunk and dense nuclear bodies by Hoechst staining, compared to non-transfected cells (Figure 5, middle panel, and white arrow). In contrast, cells transfected with mock vector or PKCδ CF-DN did not show any remarkable changes in nuclear morphology (Figure 5, upper and lower panels). These data suggest that PKCδ phosphorylates histone H3 on Ser-10 as well as histone H2B on Ser-14, triggering the chromatin condensation required for apoptotic cell death.

## Discussion

An increasing number of reports have found that post-translational modifications of histones affect overall chromatin structure and result in the regulation of nuclear function, leading to the formation of the ‘histone code’ hypothesis [29], [30]. According to this hypothesis, different combinations of histone modifications are connected to specific chromatin functions and nuclear processes during DNA repair, mitosis, meiosis, development, and apoptosis. Among the various histone modifications, specific phosphorylation sites within histone tails have been thought to affect chromatin structure and function during apoptotic cell death. It has been clearly demonstrated that phosphorylation of histones H2A, H2B, H3, and H4 is associated with nuclear events in cell death, providing cumulative evidence for an ‘apoptotic histone code’ or “death code [12], [31].” In the present study, we report that PKCδ is responsible for the phosphorylation of histone H3 on Ser-10 during apoptosis and contributes to the formation of chromatin condensation featured during programmed cell death. Therefore, this study suggests that histone H3 Ser-10 has a role in apoptosis as well as in mitosis.

The pro-apoptotic role of catalytically activate PKCδ is mediated by several downstream nuclear proteins associated with the induction of apoptosis. One of these proteins is the DNA-dependent protein kinase, an enzyme essential for the repair of double-stranded DNA breaks, which is inhibited by PKCδ-dependent phosphorylation [18]. Rad9, a key component of the genotoxin-activated checkpoint signaling complex, also binds to anti-apoptotic Bcl-2 family proteins and mediates responses to DNA damage when phosphorylated by PKCδ during apoptosis [32]. With regard to histone modification during apoptosis, it has been reported that acinus-dependent PKCδ activation increases histone H2B phosphorylation on Ser-14, which is closely associated with chromatin condensation during apoptosis [26], [33]. In addition, PKCδ also phosphorylates histone H3 on Thr-45 during apoptosis of HL60 and human neutrophil cells [34]. It has been proposed that the phosphorylation of histone H3 on Thr-45 by PKCδ causes structural changes within the nucleosome to promote DNA fragmentation during late apoptosis [12]. These recent findings collectively support the notion that activated PKCδ functions as the major histone kinase responsible for chromatin condensation and DNA fragmentation during programmed cell death. In this report, we demonstrate that PKCδ actively phosphorylates histone H3 on Ser-10 both *in vitro* and *in vivo* (Figure 1 and 2). It is noteworthy that PKCδ phosphorylates histone H3 on Ser-10 and histone H2B on Ser-14 at the same time. These results suggest that PKCδ is the histone kinase responsible for histone H3 Ser-10 phosphoryation and thus contributes to chromatin condensation in combination with other ‘death codes’ during programmed cell death.

It is extensively known that mitotic chromatin condensation is closely related to histone H3 phosphorylation on Ser-10 [35]. It is also known that there is a correlation between histone H3 Ser-10 phosphorylation and chromatin condensation during apoptosis. However, this modification has not been clearly demonstrated during cell death to date. As the phosphorylation of Ser-10 of histone H3 accompanies chromatin condensation, a similar process might occur during apoptotic cell death. We also found that activation of PKCδ was associated with the phosphorylation of histone H3 on Ser-10 during apoptosis and chromatin condensation in cells undergoing programmed cell death. Thus, it is probable that histone H3 Ser-10 phosphorylation mediates chromatin condensation during both apoptosis and mitosis. Therefore, this study provides the evidence of two distinctive functions mediated by the modification of histone H3 on Ser-10 that occurs during both apoptosis and mitosis.

**Figure 6:**
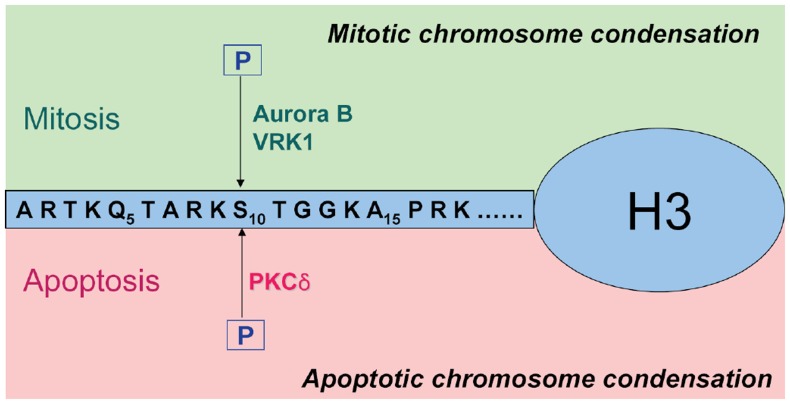
Two distinctive biological implications of the phosphorylation of histone H3 on Ser-10. In response to mitogenic stimuli, histone H3 Ser-10 is phosphorylated by mitotic kinase such as Aurora B or VRK1 to promote the mitotic chromatin condensation (upper). On the other hand, in response to death stimuli, activated PKCδ phosphorylate the same position to facilitate the apoptotic chromatin condensation (lower).

In summary, we propose the following distinctive model of the phosphorylation of histone H3 on Ser-10 both during mitosis and apoptosis (Figure 6). It is known that when cells are in a normal proliferative state, mitotic kinases such as Aurora B and VRK1 phosphorylate histone H3 on Ser-10 to promote chromatin condensation during mitosis [22], [23]. In contrast, when cells are exposed to apoptotic stimuli, activated PKCδ phosphorylates histone H3 on Ser-10 to facilitate chromatin condensation during apoptosis (Figure 6). In conclusion, histone H3 Ser-10 phosphorylation can be designated as a new ‘apoptotic histone code’ mediated by PKCδ.

## Materials and Methods

### DNA Constructs

PKCδ cloned into pHACE vector was generously given by Dr. Y. S. Lee. [2] The constitutive active form of PKCδ (amino acids 325–674, catalytic fragment, CF) was prepared by subcloning of the PCR-amplified fragment into the Xho I and Bam HI sites of pEGFP-C1 (Clontech).

### PKCδ *in vitro* Kinase Assay

Recombinant PKCδ was purchased from Upstate Biotechnology. The kinase assay was carried out in a total volume of 30 µl of a kinase reaction buffer containing 50 µM ATP, and affinity-purified GST fusion proteins or 200 µg/ml bovine purified histone H3 (Roche) as substrate for 30 min incubation at 30°C. The phosphorylated proteins were resolved on 10% SDS-polyacrylamide gels followed by immunoblot analysis.

### Preparation of Recombinant Histone H3

Synthesized oligonucleotides corresponding to histone H3 N-terminal tail (1–15) or mutated histone H3 N-terminal tail (1–15, S10A) region were cloned into pGEX-4T-3 expression vector. The resulting plasmids were transformed into *E. coli* BL21(DE3) to produce GST-histone H3 fusion proteins after treating with 0.1 M isopropyl-1-thio-β-D-galactopyranoside for 24 h at 18°C. Bacteria were lysed in a phosphate-buffered saline (PBS) containing 1 mM dithiothreitol, 1 mM PMSF, and 1 mM Na_3_VO_4_. The GST fusion proteins were then purified using glutathione-sepharose resin (GE Helathcares) and eluted from the beads with reduced glutathione according to the manufacturer’s recommendations. The mutant constructs were confirmed by DNA sequencing.

### Cell Culture and Transfection

HEK293A or HeLa cells were maintained in Dulbecco’s modified Eagle’s medium (DMEM) containing 10% fetal bovine serum and antibiotics in a humidified 5% CO_2_ incubator at 37°C. Jurkat cells were grown in RPMI 1640 containing 10% fetal bovine serum, glutamine, HEPES, and antibiotics in a humidified 5% CO_2_ incubator at 37°C. For the induction of apoptosis, cells were treated with 100 µM cisplatin (Sigma-Aldrich). For cell cycle synchronization, cells were treated with 1 mM hydroxyurea (Sigma-Aldrich) for 24 hours. HEK293A cells were transfected with the plasmid indicated in the specific experiments by using Lipofectamine 2000 (Invitrogen). After incubation for 24 h, the transfected cells were treated as indicated for analysis.

### Immunoblot Analysis and Antibodies

Immunoblot analysis was performed as we previously described. [36] Total cell lysates were incubated with anti-PKCδ (Santa Cruz Biotechnology), anti-phospho-histone H3 Ser 10 (Abcam), anti-cleaved caspase-3 (Cell Signaling), anti-histone H3 (Cell Signaling), anti-phospho histone H2B Ser 14 (Millipore), anti-phospho histone H3 Thr 3 (Cell Signalling), anti-phospho histone H3 Thr 11 (Cell Signalling) or anti-phospho histone H3 Ser 28 (Cell Signalling). Most of immunoblotting results are from three separate experiments.

### Immunocytochemical Analysis

Cells were grown on coated glass coverslips. For immunocytochemistry, cells were fixed with 4% PFA and then incubated with blocking solution (2.5% bovine serum albumin and 2.5% equine serum in PBS) for 1 h at room temperature. The samples were incubated overnight at 4°C with anti-phospho histone Ser 10 antibody (Abcam), followed by incubation with Alexa546-conjugated anti-rabbit IgG. Slides were mounted and visualized by fluorescence microscopy (Axioplan2, Zeiss; Oberkochen, Germany).

### TUNEL Assay

For the TUNEL (terminal deoxynucleotidyltransferase-mediated dUTP-biotin nick end labeling) assay, HEK293A or HeLa cells were grown on glass chips coated with poly-D-lysine (Sigma). Cells were then fixed with 4% paraformaldehyde, the glass chips were removed, and the cells were permeabilized and stained with a DeadEnd fluorometric TUNEL system kit (Promega) according to the manufacturer's instructions.

### siRNA Experiments

siRNA duplexes targeting PKCδ were purchased from Dharmacon (cat no. M-003524-01-0005). Transfection with siRNA duplexes were carried out using Lipofectamine 2000 (invitrogen) or Neon transfection system (invitrogen).

### Statistical Analysis

All quantitative data are presented as means±SD of a minimum of three experiments. Comparisons between two groups were analyzed via t test, and values of P<0.05 were considered to be significant.

## Supporting Information

Figure S1 **PKCδ also phosphorylates Ser28 of histone H3** ***in vitro.*** Recombinant PKCδ was incubated with ATP and core histone (A) or histone H3 (B). After the *in vitro* kinase(IVK) assay, the samples were analyzed by immunoblotting (IB) with anti-PKCδ or anti-phospho histone H3 Ser 28 (pH3 S28).
(TIF)

Figure S2 **Knockdown of PKCε(A) or PKCα(B) has no effect on histone H3 Ser10 phosphorylation induced by cisplatin.** Jurkat transfected with control siRNA or PKCε(A) siRNA or PKCα(B) siRNA were untreated or treated with cisplatin (50 µM) for 12 hours. The samples were analyzed by immunoblotting with indicated antibodies. The sequences of each siRNA pair were as follows: siPKCα: 5'-AAA GGC UGA GGU UGC UGA UTT-3' and 5'-AUC AGC AAC CUC AGC CUU UTT-3'; siPKCε: 5'-GCC CCU AAA GAC AAU GAA GTT-3' and 5'-CUU CAU UGU CUU UAG GGG CTT-3'.
(TIF)

Figure S3 **About 10% of TUNEL-positive cells were also positive for histone H3 Ser10 phosphorylation.** HeLa cells were treated with 1 mM hydroxyurea for 24 hours and then treated with cisplatin (50 µM) for 12 hours, and TUNEL or phospho-histone H3 Ser 10 staining cells were detected using immunofluorescence microscope.
(TIF)
