# Supplementary figures and images for: Apoptotic Phosphorylation of Histone H3 on Ser-10 by Protein Kinase Cδ
Source: PLoS One. 2012 Sep 12;7(9):e44307. doi: 10.1371/journal.pone.0044307 (PMC3440438; doi:10.1371/journal.pone.0044307)

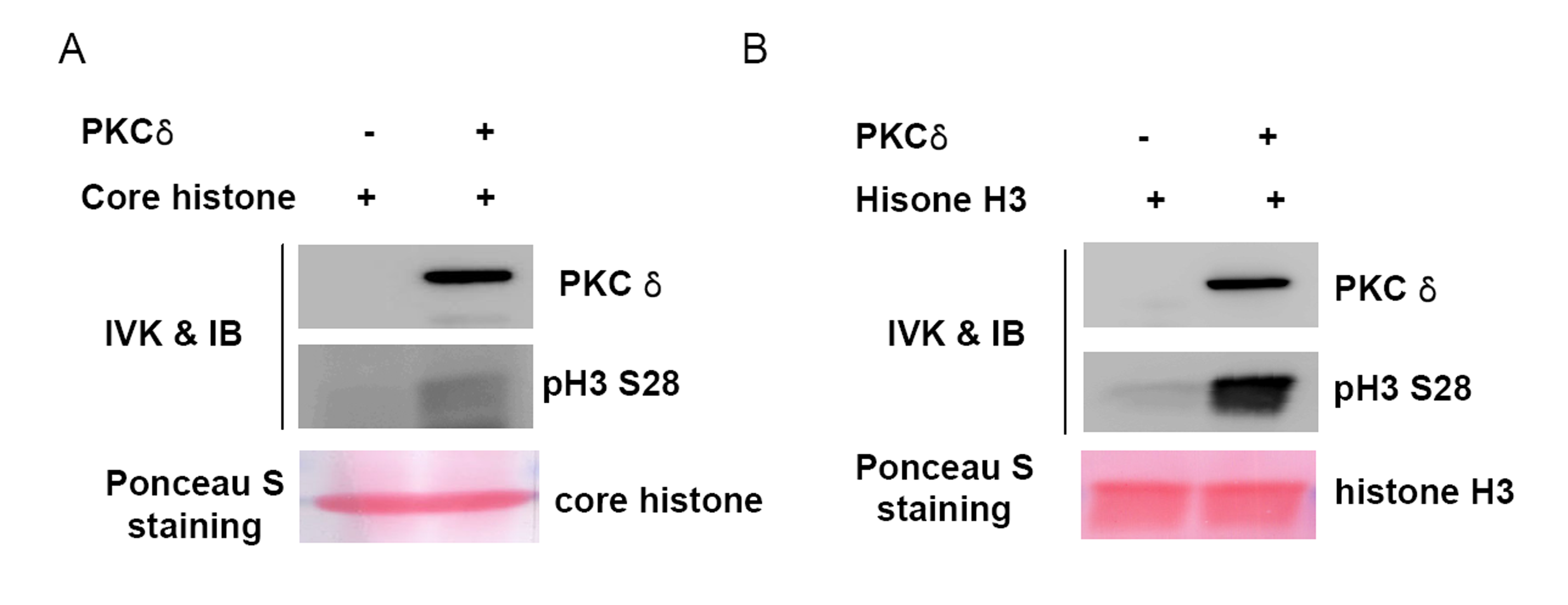

Supplement: Figure S1 — PKCδ also phosphorylates Ser28 of histone H3 in vitro. Recombinant PKCδ was incubated with ATP and core histone (A) or histone H3 (B). After the in vitro kinase(IVK) assay, the samples were analyzed by immunoblotting (IB) with anti-PKCδ or anti-phospho histone H3 Ser 28 (pH3 S28). (TIF) [file pone.0044307.s001.tif]

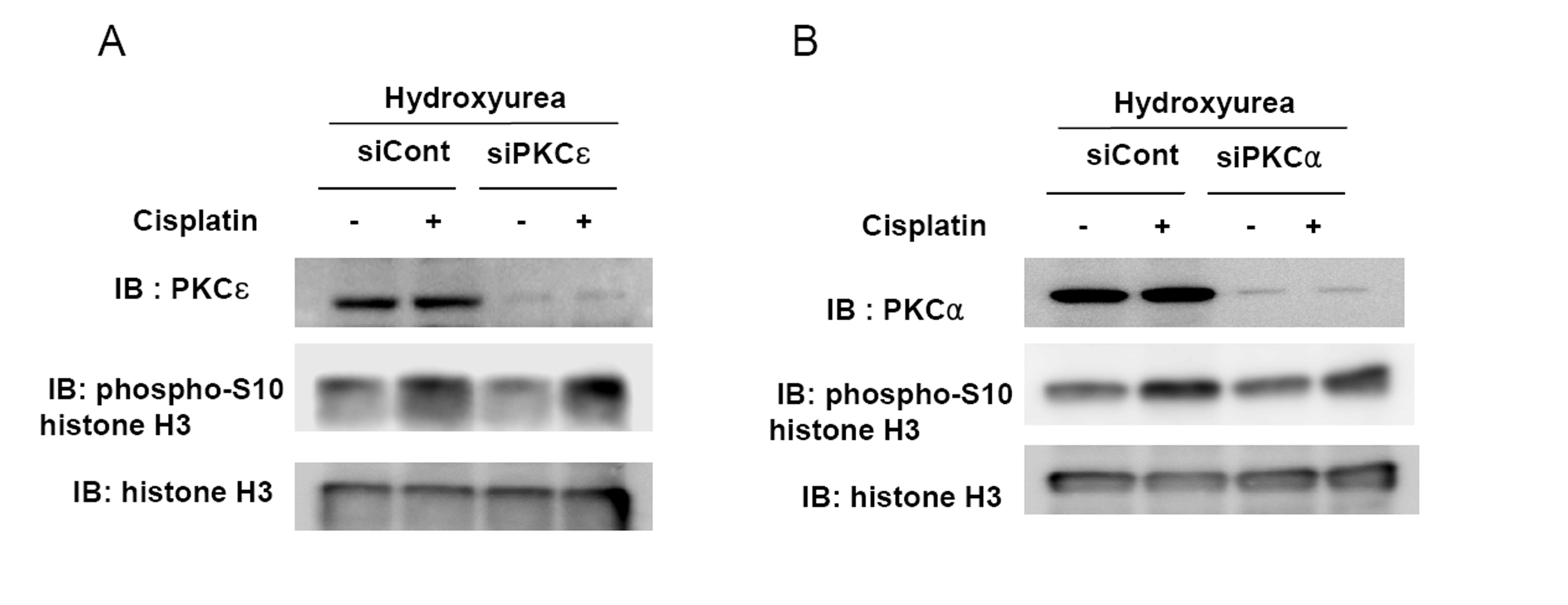

Supplement: Figure S2 — Knockdown of PKCε(A) or PKCα(B) has no effect on histone H3 Ser10 phosphorylation induced by cisplatin. Jurkat transfected with control siRNA or PKCε(A) siRNA or PKCα(B) siRNA were untreated or treated with cisplatin (50 µM) for 12 hours. The samples were analyzed by immunoblotting with indicated antibodies. The sequences of each siRNA pair were as follows: siPKCα: 5'-AAA GGC UGA GGU UGC UGA UTT-3' and 5'-AUC AGC AAC CUC AGC CUU UTT-3'; siPKCε: 5'-GCC CCU AAA GAC AAU GAA GTT-3' and 5'-CUU CAU UGU CUU UAG GGG CTT-3'. (TIF) [file pone.0044307.s002.tif]

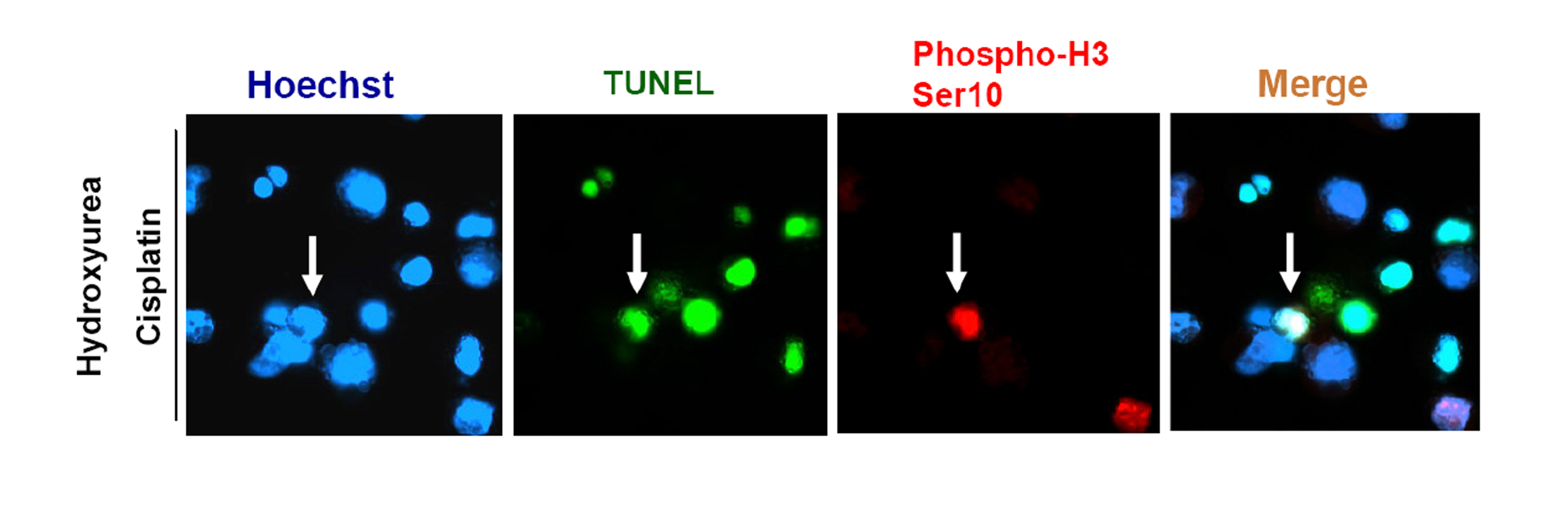

Supplement: Figure S3 — About 10% of TUNEL-positive cells were also positive for histone H3 Ser10 phosphorylation. HeLa cells were treated with 1 mM hydroxyurea for 24 hours and then treated with cisplatin (50 µM) for 12 hours, and TUNEL or phospho-histone H3 Ser 10 staining cells were detected using immunofluorescence microscope. (TIF) [file pone.0044307.s003.tif]
